# Evaluating the effectiveness of sulfidated nano zerovalent iron and sludge co-application for reducing metal mobility in contaminated soil

**DOI:** 10.1038/s41598-024-59059-7

**Published:** 2024-04-09

**Authors:** Omolola Ojo, Zuzana Vaňková, Luke Beesley, Niluka Wickramasinghe, Michael Komárek

**Affiliations:** Department of Environmental Geosciences, Faculty of Environmental Sciences, Czech University of Life Sciences Prague, Kamýcká 129, 165 00 Praha-Suchdol, Czech Republic

**Keywords:** Environmental sciences, Environmental chemistry

## Abstract

Sewage sludge has long been applied to soils as a fertilizer yet may be enriched with leachable metal(loid)s and other pollutants. Sulfidated nanoscale zerovalent iron (S-nZVI) has proven effective at metal sorption; however, risks associated with the use of engineered nanoparticles cannot be neglected. This study investigated the effects of the co-application of composted sewage sludge with S-nZVI for the stabilization of Cd, Pb, Fe, Zn. Five treatments (control, Fe grit, composted sludge, S-nZVI, composted sludge and S-nZVI), two leaching fluids; synthetic precipitation leaching procedure (SPLP) and toxicity characteristic leaching procedure (TCLP) fluid were used, samples were incubated at different time intervals of 1 week, 1, 3, and 6 months. Fe grit proved most efficient in reducing the concentration of extractable metals in the batch experiment; the mixture of composted sludge and S-nZVI was the most effective in reducing the leachability of metals in the column systems, while S-nZVI was the most efficient for reducing about 80% of Zn concentration in soil solution. Thus, the combination of two amendments, S-nZVI incorporated with composted sewage sludge and Fe grit proved most effective at reducing metal leaching and possibly lowering the associated risks. Future work should investigate the longer-term efficiency of this combination.

## Introduction

The impairment of the ecosystem services the soil provides due to contamination makes it a global problem^1^. The majority of pollution sources are from human made activities, which include mining, transportation, and land application of sewage sludge. Sewage sludge is derived as a by-product after wastewater treatment^2^. It is a mixture of water, inorganic and organic materials extracted from various wastewater sources. A significant amount of these sludges is produced globally; in the European Union alone, approximately 11 million tons of these sludges are produced annually^3^, and these sludges must be somehow used or incinerated.

Sludge must be treated to some extent before it can be further used. The type of treatment required is determined by the method of disposal. These disposal methods have great environmental significance; if not correctly disposed of, they can pose risks to public health and contaminate soil, water, and atmospheric resources; thus, adequate and proper management is critical^4^. Treatment methods change sludge properties and influence the quality of the final product. Therefore, there is a need to select the best sludge treatment, and failure to do so might have ecological, social, and economic consequences^4^. Treatment methods include aerobic and anaerobic digestion, composting, pyrolysis, thermal treatments, and stabilization with alkaline or chemical substances^5^.

Sewage sludge can be applied to land after treatment; its addition alters the soil physical, biological, and chemical properties^6^. Soil nutrient status may be improved by its application. Yet, when stored on the soil surface, sewage sludge can also damage the ecosystem because of its propensity for fermentation and hazardous compounds^7^. It contains a high concentration of metals, metalloids, pathogens, and other organic pollutants^8^. Metal(loid)s are non-biodegradable, so they can accumulate and bio-magnify in soil and sediment after their introduction^9^.

Nanotechnology has lately made it possible to design new cost-effective and environmentally friendly remediation solutions, in contrast to conventional physicochemical technologies^10^. Due to nanoparticles’ large surface area, they react more quickly with pollutants^11^. Nano zerovalent iron (nZVI) has been used as a soil stabilizing agent^12^, and it can stabilize metal(loid)s in soils. Nano zerovalent iron has long been used as an inexpensive, efficient, and environmentally friendly reductant (E^0^ =  − 0.44 V)^12^. The nZVI has also been tested for its ability to reduce and precipitate metals like Ni in biosolid thus making the metal less mobile with lower bioavailability and biotoxicity^2^. To further increase the nZVI’s reactivity and selectivity towards target pollutants, modification with sulfidation has been suggested^13,14^. This method alters nZVI with lower valent sulfur compounds, forming weakly crystalline iron sulfide in the bulk material or the nZVI particle surface^15^. The nZVI particles that have been sulfidated (S-nZVI) have demonstrated their ability to quickly remove a variety of metals, metalloids, and organic contaminants^16,17^.

Several studies have demonstrated the potential of nZVI in soil remediation^12^. However, the potential toxicological risks after nZVI application cannot be neglected. In order to minimize these unwanted side effects, the application of organic materials, including biosolids could promote plant growth and microbial activity in soils^18^. The influence of S-nZVI on the leaching of metal(loid)s in sewage sludge-amended soil using both columns and batch approaches has yet to be studied as various factors, such as wetting heat, which is the heat energy given off by dry soil when in contact with water, solid phase disturbance, shaking speed, L/S ratio, and contact time, varies in both methods may also influence how various amendments behave^19^.

The objectives of this study were to: (1) measure the beneficial effect of the co-application of sewage sludge with S-nZVI for stabilization of metal(loid)s in contaminated soils; (2) evaluate the leaching of Zn Pb, Cd and Fe from amended soils, and (3) discuss the findings in the context of environmental risks versus benefits of utilizing S-nZVI.

## Results and discussion

### Pore water

**Figure 1:**
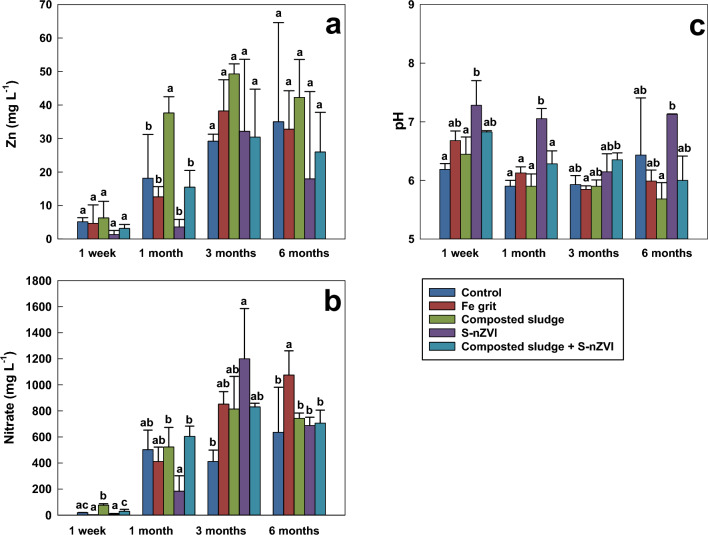
Changes in the concentration of Zn (**a**) and nitrates (**b**), and pH (**c**) in soil pore water in amended soils at different incubation times. Data with the same letter represent statistically identical values (P < 0.05) (n = 3).

The influence of amendments on metal(loid) solubility and transport was investigated using soil pore water analysis (Fig. 1). The concentrations of most elements (Pb, Cd, Fe) in pore water were below the detection limit of the ICP OES with the exception of Zn; hence, the results for Zn alone will be discussed in this section. The influence of various amendments on Zn concentration in soil solution is depicted in Fig. 1a. Composted sludge treatment caused an initial increase in the pH (6.45 ± 0.29), then reduced the pH value until the end of incubation time (5.69 ± 0.28). The initial increase in soil pH might be caused due to the presence of NH_4_-N in the sludge, and the subsequent fall was caused by an increase in nitrate concentration, as depicted in Fig. 1b. This corresponds to the findings of Kokina et al.^20^, where an initial pH increase was observed, followed by a subsequent decrease due to nitrification by the addition of N-rich residue of NH_4_-N and the synthesis of acidic by-products of decomposition. The pH decrease may also be attributed to the input of H^+^ by sewage sludge^21^.

The results show variations in the Zn concentrations after 1 week, 1 month, 3 months and 6 months. All amendments with composted sludge treatment caused an increase in the concentration of Zn in solution; however, the results were statistically insignificant at these incubation periods. The S-nZVI increases the pH after 1 month (Fig. 1c). Yan et al.^22^ also demonstrated similar Zn removal efficiency of nZVI, the main removal mechanisms for the pollutant were adsorption, complexation, and (co)precipitation^23^. The pH plays a significant role in removing Zn by nZVI; as Zn uptake increases as pH increases, this corroborates the finding of Kishimoto et al.^24^, where Zn uptake increases progressively as pH increases. The highest concentration of Zn bound to nZVI occurs at the pH of 7. Kržišnik et al.^25^ observed a similar result at pH 7, because at neutral pH, strong magnetic force enhances particle aggregation, thus increasing the potential of contaminant removal^26^.

At 1 month incubation period, composted sludge increased the concentration of Zn in soil solution by 107%, indicating that composted sludge significantly increases the content of available Zn phases and its mobility^27^. Some studies have determined that Zn may be more biologically available and mobile in soils treated with sludge^28^. McBride^29^ also showed that adding biosolids to soil can increase the fraction of bioavailable Zn. This mobility has been observed to increase with time after sewage sludge addition to the soil since it can form soluble chelates with humified and non-humidified forms of organic matter^28^. The reduction in the concentration of Zn in collected soil solution after 1 month in samples treated with Fe grit, S-nZVI and a mixture of composted sludge and S-nZVI reached 30.6%, 80.2%, and 15.5%, respectively, when compared with the control. With S-nZVI been the most efficient in decreasing Zn concentration in soil solution.

### Batch experiments

**Figure 2:**
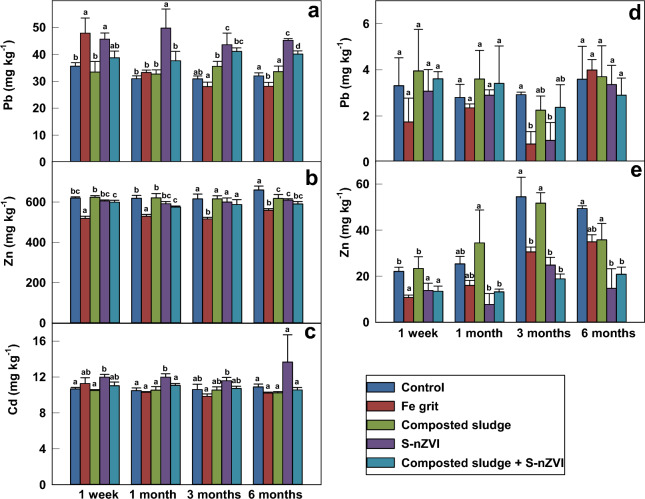
Concentration of TCLP-extractable Pb (**a**), Zn (**b**), Cd (**c**), and SPLP-extractable Pb (**d**) and Zn (**e**) after application of different amendments at different incubation times. Data with the same letter represent statistically identical values (P < 0.05) (n = 3).

The batch experiment was conducted as a controlled method to detect other metal(loid)s not detected in the soil pore water analysis. The TCLP method was used to evaluate the influence of amendments on the leaching and migration of metal(loid)s in the soil system (Fig. 2). The pH of the samples extracted by TCLP was similar for all tested samples, ranging between 4.86 and 4.96 because of the buffering nature of the TCLP solution^30^. The changes in TCLP-extractable Pb concentrations within 6 months were compared in Fig. 2a. The available Pb concentrations in the control group ranged between 30.9 ± 1.1 and 35.6 ± 1.3 mg kg^−1^ throughout the different incubation periods. The concentrations of released Pb increased in the Fe grit treated samples (47.9 ± 5.6 mg kg^−1^), and S-nZVI treated samples (45.7 ± 2.3 mg kg^−1^) after 1 week incubation period; this might be attributed to the dissolution of Fe oxides as acetic acid could increase the number of H^+^ ions that react with the –OH functional group. An increased number of positively charged Fe-OH groups could increase desorption of metal cations, which were then extracted^31^. No difference occurred between samples treated with composted sludge and a mixture of composted sludge with S-nZVI after 1 week incubation period when compared with the control samples. The concentrations of Pb extracted were reduced in Fe grit-treated samples after 1 month incubation period (33.3 ± 0.9 mg kg^−1^) when compared with control samples. A further decrease was recorded after the 3 months incubation period (28.1 ± 1.6 mg kg^−1^), indicating that Pb transformed from exchangeable to oxide-bound fraction, thus decreasing the availability of Pb with time^32^. However, there was no difference between the amount of Pb extracted in the Fe grit-treated sample after 6 months of incubation when compared with the control. S-nZVI treatment caused an increase in the amount of Pb extracted over the incubation period; this was contrary to the findings Gil-Díaz et al.^33^, where a significant reduction in the Pb extracted using TCLP occurred over a duration of 45 days. However, an earlier study by Mitzia et al.^34^ using the same contaminated soil as in our case reported an increase in exchangeable Pb fraction compared to the control after treating with nZVI. This might account for the increase in the concentration of TCLP-extracted Pb. There was no difference between the amount of Pb extracted in samples amended with a mixture of composted sludge and S-nZVI after 1 week incubation period, while a significant increase of 22%, 33% and 25% of Pb extracted after 1, 3, and 6 months incubation periods, respectively, was observed compared with the control. A slight increase in the Pb extracted was recorded for samples treated with composted sludge alone as incubation time increased, as the addition of soluble organic matter contained in composted sludge can elevate metal(loid) concentration in soil solution due to the formation of soluble organo-metal complexes^35^. Several studies also reported an increase in Pb concentration following the addition of organic material such as composted sewage sludge and composted green waste^36–38^. Overall, Fe grit caused a reduction in the concentration of extractable Pb for the first 3 months, while samples treated with S-nZVI alone and a mixture of composted sludge and S-nZVI showed an increase in the concentration of Pb extracted at most incubation times (1, 3 and 6 months).

The concentration of TCLP-extractable Zn is presented in Fig. 2b. Iron grit amendment reduces the amount of Zn extracted after 1 week, 1 month and 3 months incubation period by 16%, 14% and 16% compared with the control. Other treatments proved little to no difference in the amount of Zn extracted for the first 3 months. At the end of 6 months, all treatments caused a reduction in the amount of Zn extracted by 15%, 6%, 8%, and 10% for samples treated with Fe grit, composted sludge, S-nZVI, and a mixture of composted sludge and S-nZVI respectively.

The lowest amount of Cd extracted was from the control after 3 months (10.9 ± 0.3 mg kg^−1^) (Fig. 2c), which is about 28% of Cd leached with regards to the total concentration of Cd present in the soil sample. The S-nZVI treatment caused an increase in the amount of Cd extracted after the 1 week incubation period (12.0 ± 0.3 mg kg^−1^) when compared to the control. There was no difference between the concentration of Cd extracted in S-nZVI treated samples after 1 and 3 months, but an increase occurred after 6 months (13.7 ± 3.0 mg kg^−1^). This is contrary to the findings of Chen et al.^39^ and Xue et al.^40^, where the concentration of TCLP-extracted Cd after stabilization with a nZVI/activated carbon composite and S-nZVI, respectively, caused a decrease in the concentration of Cd extracted by TCLP. Metal speciation before and after the addition of these amendments could help to understand the mechanism of Cd stabilization, as their addition caused a decrease of exchangeable and carbonated fraction and transformation to the residual fraction in both studies. All the other amendments did not influence the concentration of Cd extracted after 6 months incubation period.

As depicted in Fig. 2d,e, all elements are released at relatively low quantities in SPLP compared to TCLP. This difference can be attributed to the extraction fluid chemistry. The TCLP fluid contains acetic acid, which can form acetate chelates, thus promoting Pb solubility^30^. The SPLP method was conducted to simulate the leaching of metal(loid)s into soil during acid rain.The pH in SPLP-extracted samples ranges between 5.95 and 6.75. In contrast to the buffering nature of TCLP, the SPLP fluid allows the sample’s pH to fluctuate^41^. The Pb concentrations extracted in all variants do not differ after the first month (Fig. 2d). At the end of the 3 months, samples treated with Fe grit and S-nZVI showed a reduction by 73% and 68% in the amount of Pb extracted, respectively, when compared to the control. The differences in the Pb concentrations in extracts from SPLP-treated samples when compared to TCLP-treated samples may be attributed to the adsorption of Pb to soil organic acids^42^. Lead could form complexes with acetate ions. These complexes would compete with the adsorption reaction of Pb ions with the surface; since hydrolyzed metal species and metal complexes are preferentially adsorbed over free metal ions, the formation of more thermodynamically favorable precipitate may cause a decrease in the concentration of dissolved Pb^42^. Chen et al.^39^ and Pinto and Al-Abed^30^ also demonstrated a similar decrease in Pb concentration in SPLP-leached sediments when compared with the TCLP-leached samples. Significant reduction in the concentration of Zn extracted from variants with Fe grit (52%, 44%, 29%), S-nZVI (38%, 55%, 70%), and a mixture of composted sludge and S-nZVI treated samples (39%, 65%, 59%) was observed after 1 week, 3 months and 6 months incubation periods, respectively, when compared with the control (Fig. 2e). No difference was observed between the samples treated with composted sludge alone at all incubation periods compared to the control.

### Column leaching

#### TCLP

**Figure 3:**
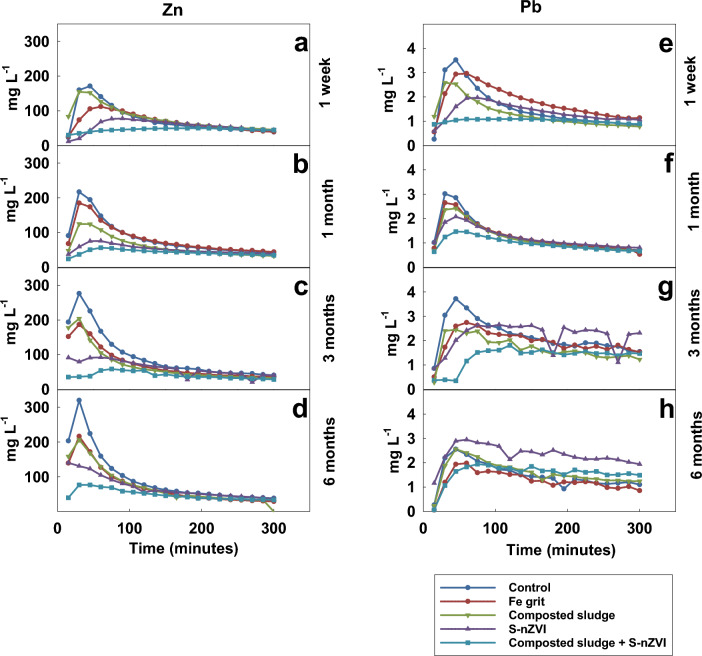
The concentration of Zn, Pb, leached by the TCLP in the column experiment after the application of various soil amendments at different incubation times.

**Figure 4:**
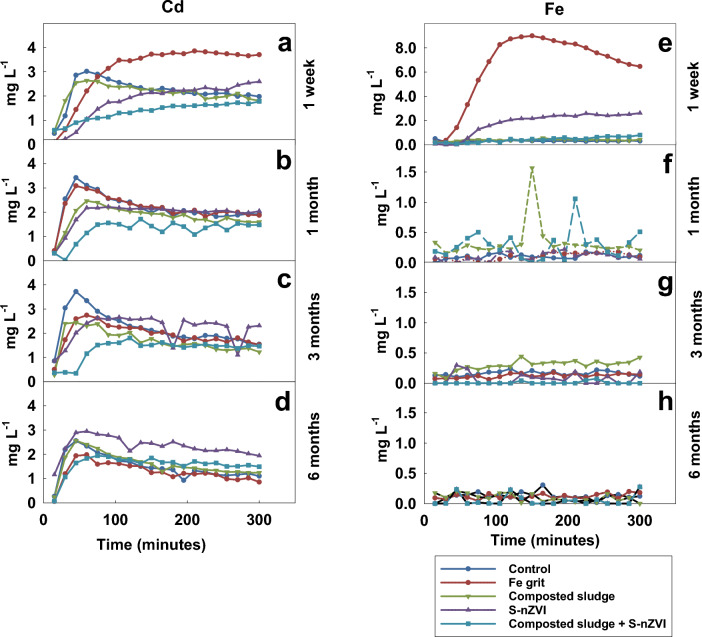
The concentration of Cd, Fe leached by the TCLP in the column experiment after the application of various soil amendments at different incubation times.

Column experiment was performed to evaluate the effect of various amendments on the mobility of metal(loid)s under dynamic flow conditions in contrast to the static batch experiment. The changes in the concentration of Zn and Pb, and Cd and Fe leached using TCLP are depicted in Figs. 3 and 4 below. The most significant differences among the amendments were observable within the first 120 min, while the system reached the equilibrium roughly after 120 min, except in samples treated with a mixture of S-nZVI and composted sludge, which caused a continuous decrease in the concentrations of most elements, throughout all sampling times when compared with the control. The Fe grit causes an increase in the concentration of Cd (Fig. 4a) and Pb (Fig. 3e) leached after the first 75 min at the end of 1 week incubation period when compared with the control; this effect was short-lived as the concentration of Zn (Fig. 3b), Pb (Fig. 3f), and Cd (Fig. 4b) leached after 1 month incubation was no different when compared with the control. However, Fe grit caused a reduction in the concentration of Cd, Zn and Pb up to the first 120 min after 3 months and 6 months incubation period in comparison with the control, with Fe grit being the best amendment for reduction of Pb leaching at the end of the 6 month incubation period. Leachates from columns containing soils treated with S-nZVI had much lower Pb (up to 1 month) and Zn (up to 6 months) contents than those from control soils. The immobilization of Zn was less effective than Pb, and this difference is attributed to the difference in chemical characteristics between the two metals. The removal mechanism for Pb by nZVI is sorption and partial chemical reduction, Zn ions are bound to Fe surface by sorption or surface complex formation^43^. Gil-Díaz et al.^44^ reported a similar decrease in the concentrations of Pb and Zn in leachates from columns containing soils treated with nZVI, being significantly lower than those from the untreated soils.

The S-nZVI amendment increased the concentration of Cd towards the end of sampling time (105 min) after 1 week (Fig. 4a) and 3 months (Fig. 4c) incubation period when compared with the control. Composted sludge alone had little to no influence on the change in concentration of Zn and Pb at the end of 1 week of incubation. However, both Pb and Zn concentrations were significantly reduced after 1 month and 3 months, after which the effect phased out and at 6 months, as no difference was observed compared to the control. Several studies investigating the Zn mobility changes driven by sewage sludge application found that the adsorption of Zn to the soil solid phase caused a reduction in the amount of Zn leached in columns^21,45^, indicating that soil solid surface acted to remove soluble Zn from its organic ligand. Zinc can also be bound to organic matter or carbonates present in sewage sludge. The effect of the mixture of composted sludge and S-nZVI treatment was pronounced throughout all incubation periods as the most effective amendment for the reduction of the leaching of all elements (Zn, Cd, and Pb) at all sampling times. However, after 6 months, this amendment increases the concentration of Pb (Fig. 3h) and Cd (Fig. 4d) when compared with the control sample towards the end of the sampling time.

#### SPLP

**Figure 5:**
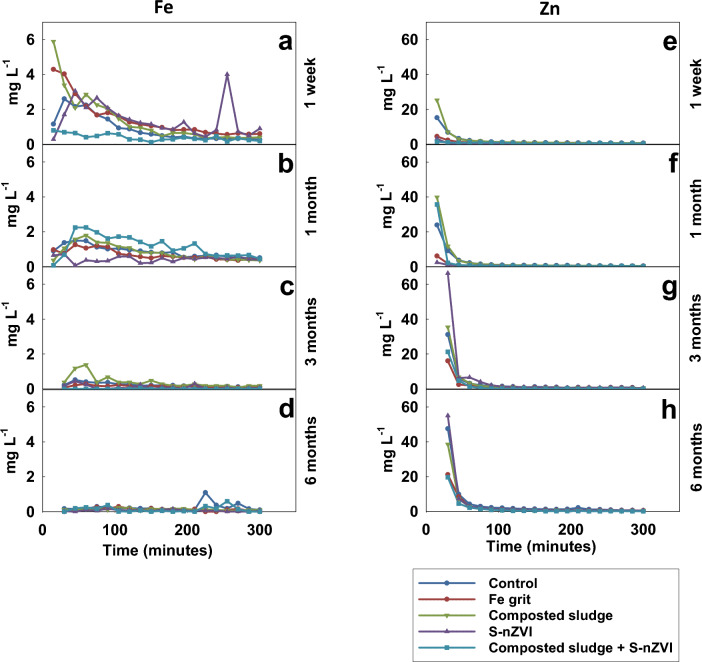
The concentration of Fe and Zn leached from the columns with amended soils using SPLP at different incubation times.

The concentration of Zn and Fe leached from amended soil using SPLP is depicted in Fig. 5 below. All amendments’ influence on Zn leaching is pronounced mainly in the first 45 min at all incubation times. It suggests that the SPLP-leached samples reach equilibrium faster than the TCLP-leached samples. The concentrations of Cd and Pb leached using SPLP were below the detection limit of ICP OES, indicating the effect of the fluid chemistry on the leaching of these metals. For instance, acetic acid present in TCLP can promote Pb leaching due to the formation of soluble acetate chelates^30^.

Composted sludge treatment increases the concentration of Zn only in the first 15 min at all incubation periods, after which the concentration of Zn leached does not differ from control. S-nZVI reduces Zn leaching after the first 45 min after 1 week and 1 month incubation period but increases Zn leaching at the first sampling time (15 min), after which no difference occurs when compared with the control. The Fe grit reduces Zn leaching only in the first 15 min at all incubation periods, after which concentrations do not differ compared to the control. Samples treated with a mixture of composted sludge and S-nZVI reduce Zn leaching only in the first 60 min at all incubation times compared with the control.

The influence of applied amendments on Fe leaching is depicted in Fig. 5. The addition of all amendments increases the concentration of released Fe at 1 week variants, with the exception of samples treated with a mixture of composted sludge and S-nZVI. The S-nZVI and Fe grit amendment reduced Fe concentration at 1 month, but samples treated with a mixture of composted sludge and S-nZVI caused an increase in Fe concentration in the same incubation period. No difference occurs between Fe concentrations after 3 and 6 months in all amendments. However, most of the Fe concentrations leached from samples with composted sludge and S-nZVI mix were below the detection limit of ICP OES at most sampling points for 3 and 6 months.

The differences in the effect of amendments in the batch and column system may be attributed to the discrepancy in the experimental method as variations exist between them, certain factors like shaking speed, L/S ratio, and contact time are not the same in both experiments and may also impact the behavior of amendment.

### Benefits and risk of S-nZVI usage

The use of S-nZVI for the stabilization of metal(loid)s appears to be a promising option. S-nZVI has been described in recent years to improve nanoparticle selectivity and reaction rate for target pollutants. While the use of S-nZVI may be considered as the ‘best’ treatment option in some cases, it might not be suitable in other cases; as obtained from our study, the application of S-nZVI alone without co-application of composted sludge was the best for the removal of Zn from soil solution. For the batch experiment, S-nZVI caused an increase in the concentration of Pb for TCLP-leached samples. The co-application of S-nZVI and composted sludge in the column experiment resulted in a constant decline in all elements of interest; therefore, it is possible to infer that several factors, including the experimental setup, may affect S-nZVI efficiency. Besides adsorption/surface complexation, another reaction mechanism between metallic contaminants and S-nZVI is sulfide precipitation, since sulfur has a large affinity for metals. Sulfur is also oxidized to S^0^ and SO_4_^2−^ during the reaction and the environmental impact of the S-nZVI is thus of concern^46^.

Rank abundance diversity analysis indicates that S-nZVI treatment poses lower cytotoxicity to microbial diversity. Additionally, microbial community responses after S-nZVI treatment showed that proteobacteria were promoted while actinobacteria were repressed^47^. Semerád et al.^48^ investigated the effects of S-nZVI on an activated sludge microbial community and showed no discernible negative effects, which was in contrast to the findings of Hui et al.^49^, where S-nZVI increased soil microbial diversity and altered the structure of bacterial and fungal communities. Cheng et al.^50^ also used *Escherichia coli* as a model organism to assess the toxicity of S-nZVI in an aqueous environment and indicated that S-nZVI had low toxicity (less than 0.35-log) to *E. coli*, indicating that there was no risk associated with S-nZVI in aquatic environments.

## Materials and methods

### Characterization of tested soil and amendments

The soil sample used in this study was obtained from the smelting region of Pribram, Czech Republic. The Pribram soil has been polluted from metallurgical activities through atmospheric deposition of contaminants and by historical floods, which enriched the soil with risk metal(loid)s. According to earlier studies conducted in the study area, Pb, Cd, and Zn are mainly prevalent in the most mobile fractions^51^. Even though mining was stopped in 1978, the smelting activities are still operational.

The soil’s top horizon (0–20 cm) was taken, air-dried, homogenized, and sieved through a 2 mm sieve. The soil was already characterized by Michálková et al.^52^. The S-nZVI slurry used was produced by Nano Iron Ltd. (NANOFER 25DS, Czech Republic), and the Fe grit was provided as a waste product originating from cast iron cutting. The composted sludge was obtained from a wastewater treatment plant in the Czech Republic. Various composting piles were set up, and the biomass for composting was prepared by mixing raw sewage sludge and grass pruning. The composting duration was eight months. Total elemental concentrations in the composted sludge and Fe chips were determined using ICP OES (iCAP 7000 series, ICP spectrometer, Thermo Scientific, USA) after microwave acid digestion which was performed following US EPA method 3050A using a representative 0.5 g of sample in a mixture of HNO_3_, HCl and HF (9:3:1). Analytical blanks containing only the reagent were also prepared. After the microwave digestion, samples were evaporated to dryness at 100 °C and dissolved in 10 mL 2% HNO_3_. Quality control of sample digestion was done by analyzing certified reference materials BCR-483-70G and NIST2710A and comparing the results obtained with the certified values. The physicochemical properties of the tested contaminated soil and soil amendments are summarized in Table 1.

**Table 1:** The physicochemical characteristic of the study soil represented as mean ± standard deviation (n = 3).

pH_H2O_	5.95 ± 0.02		
pH_KCl_	5.14 ± 0.04		
CEC (cmol/kg)	9.08 ± 0.52		
TOC (%)	2.15 ± 0.05		
Particle size distribution (%)			
Clay	Silt	Sand	
7	31	62	
Soil classification			
Sandy loam			

Total metal(loid) concentrations mg/kg (n = 3)			
mg kg^−1^		Composted sludge	Fe grit
As	296 ± 60	< dl	< dl
Pb	3539 ± 37	28.3 ± 3.4	42.2 ± 8.8
Cd	39 ± 10	< dl	< dl
Zn	4002 ± 68	358 ± 10	1226 ± 86
Cu	68 ± 30	109 ± 2	1392 ± 59
Fe	37,408 ± 19	17,592 ± 414	817,888 ± 65,610
Mn	4276 ± 34	386 ± 3	7369 ± 477

< dl results lower than the detection limit of the ICP OES.

### Soil pore water analyses

The impact of the amendments and incubation period on the chemistry of soil pore water was investigated. Five treatments were used: (control (C), 1% S-nZVI (CS), 1% Fe grit (CF) (w/w), 1% composted sludge (CC) (w/w), 1% composted sludge (w/w) + 1% S-nZVI (CCS) (w/w)). The 200 g of dry soil was used per pot with three replicates per amendment and per each incubation time (1 week, 1 month, 3 months and 6 months). Rhizon sampler (Rhizosphere Research151 Products, Netherlands) was attached to the base of each container. The pots were watered twice weekly to maintain the moisture level at the field capacity. After each incubation period, the soil pore water was collected using the Rhizon sampler and analyzed. The contents of metal(loid)s, DOC, and anions were determined as described above, and pH and Eh values were recorded.

### Batch experiment

To find out how the addition of the tested amendments affects the mobility of metal(oid)s in the contaminated soils, incubation batch experiments were conducted. For this experiment, the same amendments (type of amendment and dosage) and experimental design (incubation times, number of replicates) as in the experiment dealing with soil pore water sampling were used (see “Soil pore water analyses” section). At the end of each incubation period, soil samples were dried at 60 °C for 48 h. 2 g of the dried soil were transferred into an extraction vessel. Appropriate volumes of the SPLP solution, pH 5.00 (US EPA methods 1312), or TCLP solution, pH 4.93 ± 0.05 (US EPA method 1311), were added to soil at a 1:20 (w/v). The vessel was left to agitate for 18 h, after which samples were centrifuged at 5000 rpm for 10 min. The liquid and solid phases were separated by filtration through a 0.45 μm cellulose acetate syringe filter. The leachates collected were analyzed for metals, anions, and dissolved organic carbon; the pH and Eh values were also measured.

### Column experiment

The influence of various amendments on the transport behavior of contaminants in the tested soils was investigated using a column experiment. Similar amendment to soil pore water sampling is used (see “Soil pore water analyses” section). Five treatments were used: (control (C), 1% S-nZVI (CS), 1% Fe grit (CF) (w/w), 1% composted sludge (CC) (w/w), 1% composted sludge (w/w) + 1% S-nZVI (CCS) (w/w)). Prior to the leaching of soil samples in the columns, soils with applied amendments (including control soil) were pre-incubated for different time intervals to enable the evaluation of the time-dependent changes in contaminants’ leachability. For this purpose, a mass of 30 g of soil (amended or control) was put into the pot (this mass was recalculated depending on the percentage of each amendment). The pots were regularly watered with deionized water to maintain field capacity for different time rates (1 week, 1 month, 3 months, and 6 months). After each incubation period, the soil sample was used in the column experiment.

Two different solutions: (i) synthetic precipitation leaching procedure (SPLP) at pH 5.00 (US EPA method 1312) and (ii) toxicity precipitation leaching procedure (TCLP) at pH 4.93 ± 0.05 (US EPA method 1311) were used as leaching solutions. The TCLP aims to simulate contaminant leaching in municipal landfills. The SPLP, on the other hand, tries to mimic the leaching of contaminants under acidic precipitation. After each incubation period, samples were transferred into a column (BIO-RAD Econo glass chromatography columns of dimension 2.5 × 30 cm). For practical reasons, the columns were operated in a down-flow mode. In order to minimize the risk of preferential flow and channeling, a low flow rate (0.5 mL min^−1^) controlled using peristaltic pump was used. The same operation mode was used for all the treatments. The leachates collected were analyzed for metal(loid)s using ICP OES, anions using the ion chromatography (IC, Dionex 5000+, Thermo Fisher Scientific, USA), and the dissolved organic carbon (DOC) using a TOC/TC analyzer (TOC-LCPH, Shimadzu, Japan). The pH and Eh values were recorded.

### Statistical analysis

Statistical analyses were performed using SigmaPlot 14.0 (Statsoft Inc., USA). Experimental data were evaluated using analysis of variance (ANOVA) at P < 0.05 using the Tukey post hoc test.

## Conclusion

Sulfidated nano zerovalent iron (S-nZVI) was used in combination with composted sewage sludge to evaluate its ability to reduce the leachability of Pb, Cd, Zn, while the release of Fe from added amendments was investigated as well. In batch experiments, Fe grit amendment proved to be generally the most effective for reducing the release of targeted contaminants. In contrast, the composted sludge and S-nZVI mix amendment proved to be the most effective in column experiments. The Pb, Cu, and Zn concentrations in the TCLP extract followed the decreasing order of Zn > Pb > Cd. Differences in fluid chemistry of the leaching solution SPLP and TCLP also affect the leaching of Zn, Cd, and Pb. In common with many, or even most, studies investigating metal(loid) leaching pH is the most important factor for Zn removal by nZVI in soil solution. Both amendments (Fe grit and a mixture of composted sludge and S-nZVI) have proven effective depending on the experimental condition, with Fe grit being the most cost-efficient of the two amendments and might be a good use for waste material.

As a next step, an evaluation of the long-term effects of both amendments should be conducted. Also, the toxicological effects of both amendments should be evaluated on a short and long-term basis. Since samples amended with composted sludge alone have little to no influence in the stabilization of contaminants both in the batch and column experiment in this study, it is recommended that subsequent studies should use higher doses greater than 1% to test its stabilization potential. The obtained results show that S-nZVI is efficient for the stabilization of metal(loid)s. However, it is necessary to investigate further its fate and potential toxicity as current toxicological data are mostly derived from short-term experiments. To further comprehend any potential environmental concern, it is necessary to explore the long-term evaluation of S-nZVI especially in the field. Lastly, we advise using both short- and long-term environmental monitoring, focusing on the ecotoxicological impacts of S-nZVI as well as its transport and persistence.

## Data Availability

The datasets generated and analysed during the current study are available from the corresponding author on reasonable request.

## References

[1] Usman K (2012). Sewage sludge: An important biological resource for sustainable agriculture and its environmental implications. Am. J. Plant Sci..

[2] Li XQ, Brown DG, Zhang WX (2007). Stabilization of biosolids with nanoscale zero-valent iron (nZVI). J. Nanopart. Res..

[3] Đurđević D, Žiković S, Blecich P (2022). Sustainable sewage sludge management technologies selection based on techno–economic–environmental criteria: Case study of Croatia. Energies.

[4] Rorat A, Courtois P, Vandenbulcke F, Lemiere S, Prasad MN, de Campos Favas PJ, Vithanage M, Mohan SV (2019). Sanitary and environmental aspects of sewage sludge management. Industrial and Municipal Sludge: Emerging Concerns and Scope for Resource Recovery.

[5] Wu J (2017). Effects of thermal treatment on high solid anaerobic digestion of swine manure: Enhancement assessment and kinetic analysis. Waste Manag..

[6] Lonova K (2022). Microwave pyrolyzed sewage sludge: Influence on soil microbiology, nutrient status, and plant biomass. Chem. Biol. Technol. Agric..

[7] Boudjabi S, Chenchouni H (2021). On the sustainability of land applications of sewage sludge: How to apply the sewage biosolid in order to improve soil fertility and increase crop yield?. Chemosphere.

[8] Fijalkowski K, Rorat A, Grobelak A, Kacprzak MJ (2017). The presence of contaminations in sewage sludge—The current situation. J. Environ. Manag..

[9] Melake BA, Endalew SM, Alamirew TS, Temesegen LM (2023). Bioaccumulation and biota-sediment accumulation factor of metals and metalloids in edible fish: A systematic review in Ethiopian surface waters. Environ. Health Insights.

[10] Hidangmayum A (2022). Mechanistic and recent updates in nano-bioremediation for developing green technology to alleviate agricultural contaminants. Int. J. Environ. Sci. Technol..

[11] Roy A, Sharma A, Yadav S, Jule LT, Krishnaraj R (2021). Nanomaterials for remediation of environmental pollutants. Bioinorg. Chem. Appl..

[12] Galdames A, Ruiz-Rubio L, Orueta M, Sánchez-Arzalluz M, Vilas-Vilela JL (2020). Zero-valent iron nanoparticles for soil and groundwater remediation. Int. J. Environ. Res. Public Health.

[13] Brumovský M (2020). Core-shell fe/fes nanoparticles with controlled shell thickness for enhanced trichloroethylene removal. ACS Appl. Mater. Interfaces.

[14] Fan D (2017). Sulfidation of iron-based materials: A review of processes and implications for water treatment and remediation. Environ. Sci. Technol..

[15] Dong H (2018). Factors influencing degradation of trichloroethylene by sulfide-modified nanoscale zero-valent iron in aqueous solution. Water Res..

[16] Rajajayavel SRC, Ghoshal S (2015). Enhanced reductive dechlorination of trichloroethylene by sulfidated nanoscale zerovalent iron. Water Res..

[17] Xu J (2019). Reactivity, selectivity, and long-term performance of sulfidized nanoscale zerovalent iron with different properties. Environ. Sci. Technol..

[18] Muter O, Dubova L, Kassien O, Cakane J, Alsina I (2022). Application of the sewage sludge in agriculture: Soil fertility, technoeconomic, and life-cycle assessment. Hazard. Waste Manag..

[19] Reyhanitabar A, Ramezanzadeh H, Oustan S, Neyshabouri M (2017). Comparison of batch and column methods in zinc sorption in a sandy soil. Int. J. Adv. Sci. Eng. Technol..

[20] Kokina K (2022). Impact of rapid pH changes on activated sludge process. Appl. Sci..

[21] Jalali M, Arfania H (2010). Leaching of heavy metals and nutrients from calcareous sandy-loam soil receiving municipal solid sewage sludge. J. Plant Nutr. Soil Sci..

[22] Yan W, Herzing AA, Kiely CJ, Zhang WX (2010). Nanoscale zero-valent iron (nZVI): Aspects of the core-shell structure and reactions with inorganic species in water. J. Contam. Hydrol..

[23] Liang W, Dai C, Zhou X, Zhang Y (2014). Application of zero-valent iron nanoparticles for the removal of aqueous zinc ions under various experimental conditions. PLoS ONE.

[24] Kishimoto N, Iwano S, Narazaki Y (2011). Mechanistic consideration of zinc ion removal by zero-valent iron. Water Air Soil Pollut..

[25] Kržišnik N (2014). Nanoscale zero-valent iron for the removal of Zn2+, Zn(II)-EDTA and Zn(II)-citrate from aqueous solutions. Sci. Total Environ..

[26] Nik Redzauddin NNI, Kassim J, Amir A (2015). Removal of zinc by nano-scale zero valent iron in groundwater. Appl. Mech. Mater..

[27] Bowszys T, Wierzbowska J, Sternik P, Busse MK (2015). Effect of the application of sewage sludge compost on the content and leaching of zinc and copper from soils under agricultural use. J. Ecol. Eng..

[28] Zaragüeta A (2021). Effect of the long-term application of sewage sludge to a calcareous soil on its total and bioavailable content in trace elements, and their transfer to the crop. Minerals.

[29] McBride MB (2022). Long-term biosolids application on land: Beneficial recycling of nutrients or eutrophication of agroecosystems?. Soil Syst..

[30] Pinto PX, Al-Abed SR (2017). Assessing metal mobilization from industrially lead-contaminated soils located at an urban site. Appl. Geochem..

[31] Danila V, Janusevicius T (2022). Removal of Cd, Cu, Ni, and Pb from nanoscale zero-valent iron amended soil using 0.1 M acetic acid solution. Environ. Clim. Technol..

[32] Parvin A (2022). Chemical speciation and potential mobility of heavy metals in organic matter amended soil. Appl. Environ. Soil Sci..

[33] Gil-Díaz M, López LF, Alonso J, Lobo MC (2018). Comparison of nanoscale zero-valent iron, compost, and phosphate for Pb immobilization in an acidic soil. Water Air Soil Pollut..

[34] Mitzia A, Vítková M, Komárek M (2020). Assessment of biochar and/or nano zero-valent iron for the stabilisation of Zn, Pb and Cd: A temporal study of solid phase geochemistry under changing soil conditions. Chemosphere.

[35] Zhou YF, Haynes RJ (2010). Sorption of heavy metals by inorganic and organic components of solid wastes: Significance to use of wastes as low-cost adsorbents and immobilizing agents. Crit. Rev. Environ. Sci. Technol..

[36] van Herwijnen R (2007). Remediation of metal contaminated soil with mineral-amended composts. Environ. Pollut..

[37] Bolan N (2014). Remediation of heavy metal(loid)s contaminated soils—To mobilize or to immobilize?. J. Hazard. Mater..

[38] Schwab P, Zhu D, Banks MK (2007). Heavy metal leaching from mine tailings as affected by organic amendments. Bioresour. Technol..

[39] Chen W-F, Wang W, Zhang X, Zhang J (2016). Stabilization of heavy metals in contaminated river sediment by nanozero-valent iron/activated carbon composite. J. Environ. Eng..

[40] Xue W (2023). Immobilization of cadmium in river sediments using sulfidized nanoscale zero-valent iron synthesized with different iron precursors: Performance and mechanism. J. Soils Sedim..

[41] Dungan RS, Dees NH (2009). The characterization of total and leachable metals in foundry molding sands. J. Environ. Manag..

[42] Al-Abed SR, Hageman PL, Jegadeesan G, Madhavan N, Allen D (2006). Comparative evaluation of short-term leach tests for heavy metal release from mineral processing waste. Sci. Total Environ..

[43] Li XQ, Zhang WX (2007). Sequestration of metal cations with zerovalent iron nanoparticles—A study with high resolution X-ray photoelectron spectroscopy (HR-XPS). J. Phys. Chem. C.

[44] Gil-Díaz M (2014). Immobilization and leaching of Pb and Zn in an acidic soil treated with zerovalent iron nanoparticles (nZVI): Physicochemical and toxicological analysis of leachates. Water Air Soil Pollut..

[45] Ashworth DJ, Alloway BJ (2004). Soil mobility of sewage sludge-derived dissolved organic matter, copper, nickel and zinc. Environ. Pollut..

[46] Liang L (2021). The removal of heavy metal cations by sulfidated nanoscale zero-valent iron (S-nZVI): The reaction mechanisms and the role of sulfur. J. Hazard. Mater..

[47] Liu N (2023). Sulfidated nanoscale zero valent iron for in situ immobilization of hexavalent chromium in soil and response of indigenous microbes. Chemosphere.

[48] Semerád J (2020). Environmental fate of sulfidated nZVI particles: The interplay of nanoparticle corrosion and toxicity during aging. Environ. Sci. Nano.

[49] Hui C (2022). Transformation of sulfidized nanoscale zero-valent iron particles and its effects on microbial communities in soil ecosystems. Environ. Pollut..

[50] Cheng Y (2024). Elucidating the impact of sulfur precursors on the reactivity, toxicity, and colloidal stability of post-sulfidized nanoscale zerovalent iron. Sep. Purif. Technol..

[51] Nováková T (2015). Pollutant dispersal and stability in a severely polluted floodplain: A case study in the Litavka River, Czech Republic. J. Geochem. Explor..

[52] Michálková Z, Komárek M, Vítková M, Řečínská M, Ettler V (2016). Stability, transformations and stabilizing potential of an amorphous manganese oxide and its surface-modified form in contaminated soils. Appl. Geochem..

